# Anterior Hip Dislocation After Hip Arthroscopy Complicated by Iliopsoas Bursitis

**DOI:** 10.7759/cureus.17044

**Published:** 2021-08-10

**Authors:** Matthew H Nasra, Christopher R Michel, Suleiman Sudah, Christopher Dijanic, Brian Torpey

**Affiliations:** 1 Orthopedic Surgery, Rutgers Robert Wood Johnson Medical School, New Brunswick, USA; 2 Orthopedic Surgery, Monmouth Medical Center, Long Branch, USA

**Keywords:** dislocation, arthroscopy, bursitis, hip, iliopsoas

## Abstract

Hip dislocation after hip arthroscopy is an uncommon postoperative complication. We report a case of a 51-year-old woman who underwent right hip arthroscopy and presented with an anterior hip dislocation on postoperative day five. The index surgery involved capsulotomy, cam lesion debridement, and femoroplasty for an anterosuperior labral tear and cam-type femoroacetabular impingement. The patient underwent an uneventful recovery course until eight weeks postoperatively she developed iliopsoas bursitis. Her symptoms were managed conservatively with activity modification and physical rehabilitation. Complete resolution of symptoms was reported by the six-month follow-up visit, and no further dislocations or instability had been reported at 12 months. Anterior hip dislocation is a rare complication following hip arthroscopy and patients may experience persistent iliopsoas bursitis several months following successful reduction.

## Introduction

Hip arthroscopy (HA) is a surgical procedure increasing in popularity in recent years due to its relatively low complication rate and less invasive approach compared to traditional hip surgery [1,2]. Hip dislocation after HA is an infrequent complication [3-13]. Here, we report a case of an anterior hip dislocation occurring on a postoperative day five following capsulotomy, cam lesion debridement, and femoroplasty for an anterosuperior labral tear and cam-type femoroacetabular impingement (FAI). This case is unique as it is one of the first to report a dislocation in the acute postoperative period that had an association with persistent iliopsoas bursitis several weeks after successful closed reduction.

## Case presentation

The patient is a 51-year-old woman who underwent right hip arthroscopy with labral repair and femoroplasty with cam lesion debridement. After failing years of conservative management for unremitting right hip and groin pain, she elected to undergo right hip arthroscopy. Following surgery, she was made partially weight-bearing with an early range of motion to assist in recovery. She experienced an uneventful postoperative course until day five. While cleaning her kitchen, she extended and externally rotated her operative leg resulting in sudden and severe right hip pain accompanied by a fall to the ground and an inability to bear weight.

**Figure 1:**
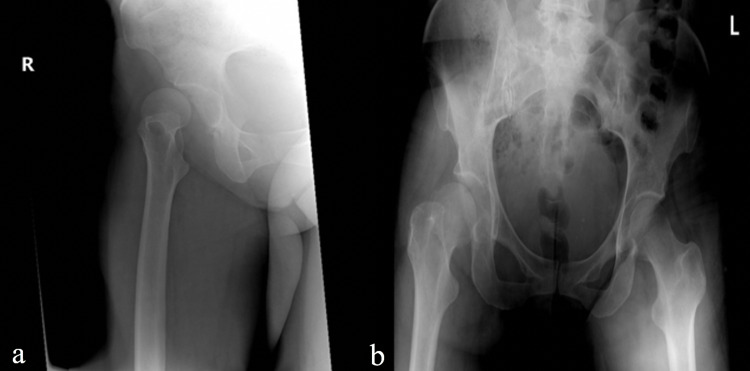
Radiographs of the right hip and pelvis showing anterior hip dislocation. (a) Anteroposterior (AP) view of the right hip. The radiograph demonstrates that the femoral head has dislocated from the acetabulum in an anterior direction. (b) AP view of the right pelvis. This radiograph also shows that the right femoral head is dislocated compared to the contralateral side.

Upon presentation, her right leg was shortened and externally rotated with intact neurovascular function. Radiographs revealed right anterior hip dislocation without evidence of acute fracture (Figure 1).

**Figure 2:**
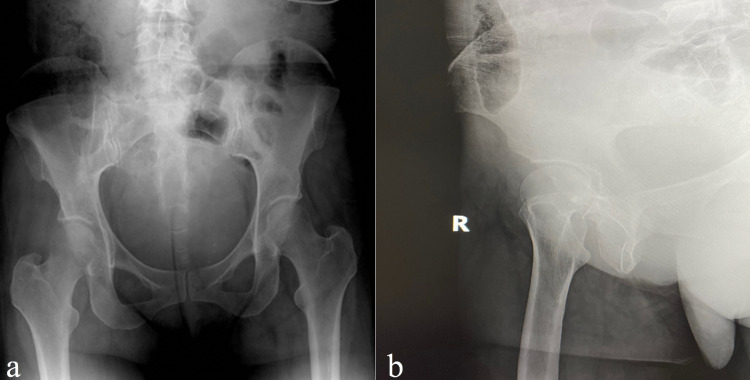
Radiographs of the right hip and pelvis postreduction. (a) Anteroposterior (AP) view of the right pelvis and (b) lateral view of the right hip. These images show a well-reduced right hip joint with the femoral head and acetabulum in appropriate alignment.

She was consciously sedated, closed reduced, and placed in a knee immobilizer (Figure 2).

**Figure 3:**
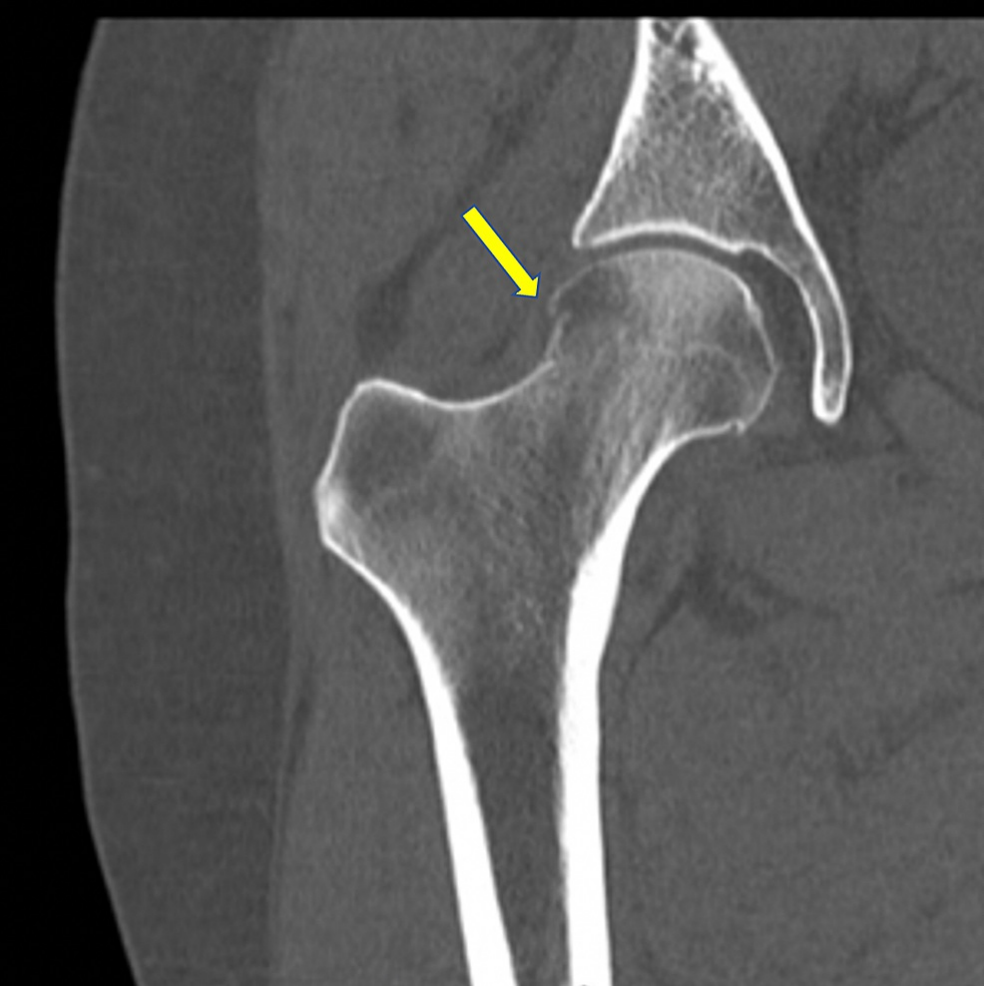
CT of the right hip. Cortical step-off (yellow arrow) suspicious for impaction-type fracture in the anterior aspect of the femoral head.

Postreduction computed tomography (CT) revealed an impaction-type fracture in the anterior aspect of the femoral head (Figure 3).

**Figure 4:**
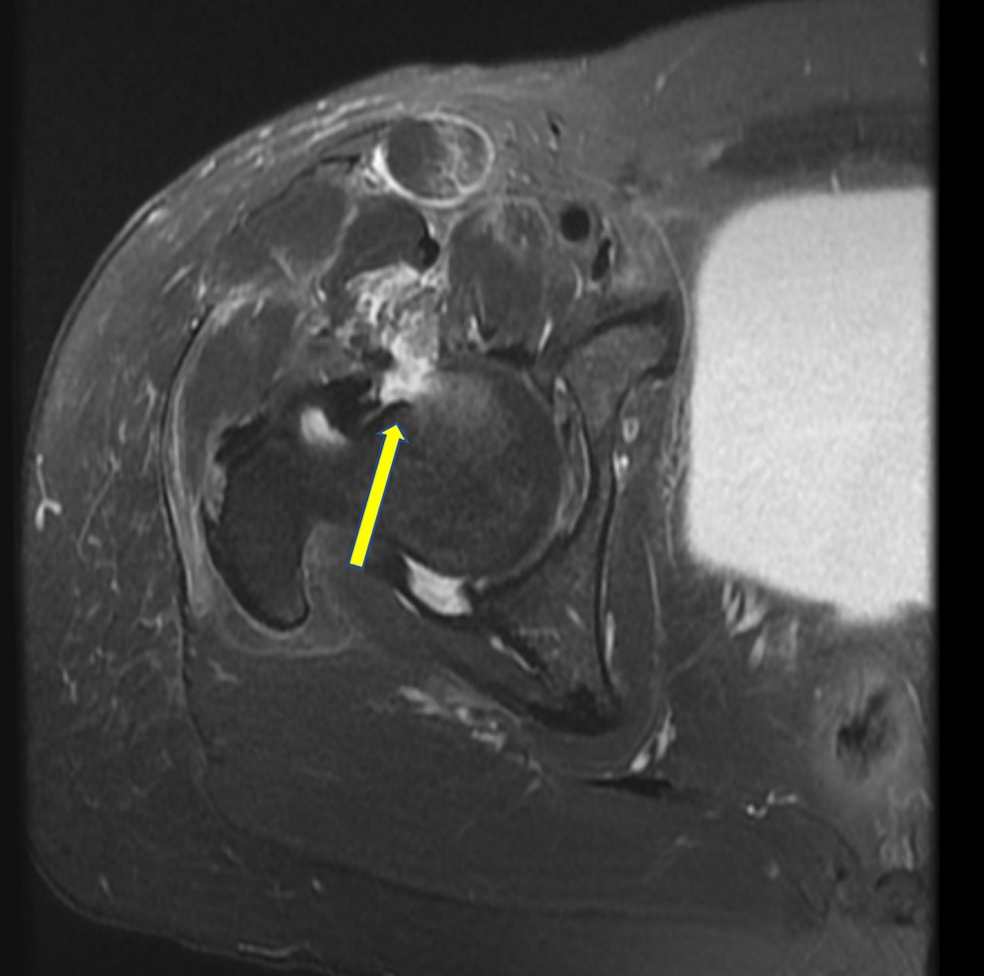
MRI of the right hip. T2-axial fat-suppressed view demonstrates edema-like signal in the anterior femoral head and neck junction suggestive of impaction fracture (yellow arrow).

Magnetic resonance imaging (MRI) was also obtained, which revealed an impaction fracture involving the anterior margin of the right femoral head and neck junction (Figure 4).

Surgical pathology of the cam resection and femoroplasty ruled out the presence of bony pathology. By one week postoperatively, she was able to ambulate with a walker. Physical examination revealed painless hip flexion greater than 100 degrees, extension to neutral, internal rotation to 35 degrees, and abduction to 45 degrees. However, she still experienced a sensation of anterior hip popping and had crepitus anteriorly. Initial MRI and CT scan were re-reviewed, and the labral repair appeared to be intact. She was advised to limit external rotation and hyperextension of the hip with protected weight-bearing and crutch use.

By six weeks postoperatively, her preoperative anterior hip popping and crepitus had entirely resolved. She experienced no pain in the anterior hip capsule and no apprehension with hyperextension or external rotation. The patient had a stable gait without the use of assistive devices and was able to perform a full crouch without difficulty.

The patient did not remain symptom-free during her continued follow-up visit. By eight weeks postoperatively, she experienced a re-intensification of her right hip pain that she characterized as a deep ache in the joint. Her hip occasionally locked and was accompanied by a sense of instability. She denied a history of trauma - other than the recent hip dislocation - or inciting events. Physical examination revealed palpable crepitus in the anterior acetabular margin at 60 degrees to 90 degrees of flexion. Both internal and external rotation aggravated these symptoms. The patient could ambulate, although she preferred to take small steps with her right lower extremity to reduce pain. Radiographs of the right hip revealed a well-maintained joint surface and joint line architecture with no gross bony abnormalities.

**Figure 5:**
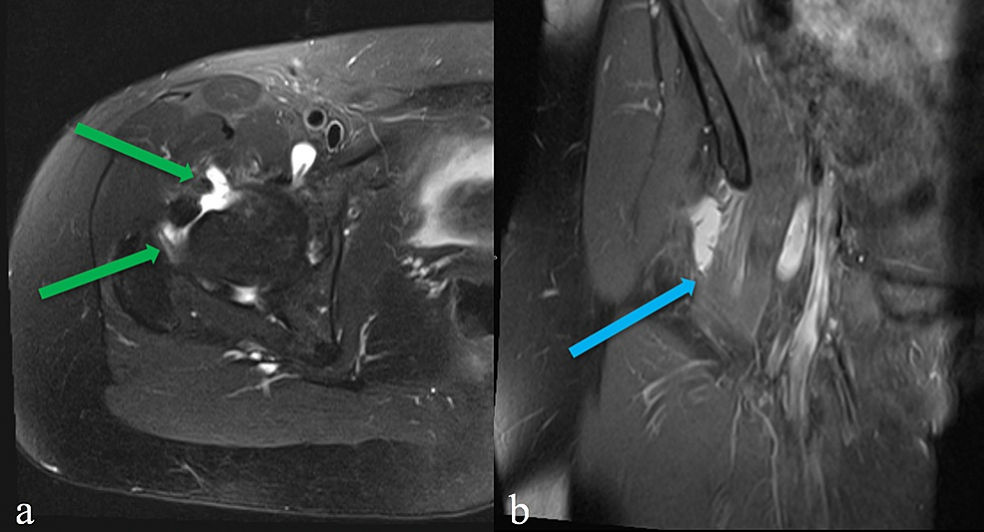
MRI of the right hip. (a) T2-axial fat-suppressed. Filling defects in the joint recesses and the iliopsoas bursa suspicious for synovial proliferation and small intra-articular bodies (green arrows). (b) Coronal proton-density (PD). Edema-like signal in the right iliopsoas muscle (blue arrow) compatible with tendinosis/tendinopathy and fluid in the iliopsoas bursa.

Subsequent MRI revealed right iliopsoas bursitis with filling defects in the joint recesses and the iliopsoas bursa suspicious for synovial proliferation and small intra-articular bodies (Figure 5).

Additionally, new-onset small focal cartilage loss and osteochondral impaction injury in the femoral head secondary to microtrauma and instability were identified. Her symptoms were managed conservatively with activity modification and physical rehabilitation. Complete resolution of symptoms was reported by a six-month follow-up, and no further dislocations or instability had been reported at 12 months.

## Discussion

HA is a low-risk, minimally invasive procedure with numerous indications, including acetabular labral tears, FAI, chondral lesions, osteochondritis dissecans, ligamentum teres injuries, snapping hip syndrome, iliopsoas bursitis, and loose bodies [2]. Hip arthroscopy is an appealing procedure to surgeons due to its low rate of major complications. A systematic analysis by Harris et al. revealed that iatrogenic chondrolabral injury is the most common complication of HA, with hip dislocations occurring far less frequently [14]. In particular, hip dislocations accounted for four out of the 6134 cases examined in their study [14]. Additionally, Larson et al. reported no cases of iatrogenic hip instability in 1026 arthroscopies reviewed [15].

With such an exceedingly low complication rate, post arthroscopic hip dislocation has not been well documented. The majority of cases of hip dislocation after HA reported have been anterior hip dislocations [3-13]. Alternatively, in the general population, anterior hip dislocations happen far less frequently than posterior dislocations [16]. Several surgical risk factors during hip arthroscopy are thought to contribute to post arthroscopic hip instability. These include failure to repair the capsule, iliopsoas debridement and tenotomy, cam resection, and partial psoas release [4]. While the surgical technique is crucial for success, it is equally important to identify contributing patient risk factors. Patient risk factors that increase the risk of dislocation following HA include female gender, ligamentous laxity, connective tissue disease, acetabular dysplasia, femoral anteversion greater than 40 degrees, a center-edge angle of fewer than 25 degrees, and an increased capsular volume [17,18].

Our patient underwent a transverse limb capsulotomy of the anterior joint capsule without repair, rim trimming of the acetabular margin, and femoroplasty with cam lesion resection. Interestingly, our patient experienced anterior hip dislocation only five days post arthroscopy. The patient also sustained a femoral head fracture, intra-articular loose bodies, and focal cartilage loss that resulted from the original injury. We can postulate from our experience that intraoperative distension and stretching of the anterior joint capsule may create instability and thus the risk for dislocation and subsequent injuries. Prior studies reported dislocation events that occurred immediately in the recovery room [6], on postoperative day one [13], three weeks postoperatively [10], and the remaining between two and seven months [4,7-9,11,12].

Inciting mechanisms vary depending on the activity level of the individual. Less active individuals frequently report a low energy twist or fall [7,10,11]. Alternatively, more active individuals report participation in higher energy events such as long jumping [9], javelin throwing [8], running [8], or ballet dancing [12]. While these vary drastically, they all have a common component of hip extension and external rotation, which was also present in our case.

Our patient was diagnosed with new-onset iliopsoas bursitis eight weeks following closed reduction of her post arthroscopic anterior hip dislocation. Six other cases describe further complications that required additional surgery: residual pain and apprehension treated with arthroscopic capsular plication [7], gross hip instability treated with mini-open capsular repair [6], severe instability treated with total hip arthroplasty [13], persistent instability treated with revision open capsulorrhaphy [11], persistent instability treated with capsular reconstruction [12], and progressive end-stage arthritis treated with total hip arthroplasty [10]. The remaining cases discuss patients who experienced a baseline return to function with no long-term complications [8,9].

## Conclusions

Anterior hip dislocation after HA is an uncommon postoperative complication of an otherwise safe and well-regarded procedure. Surgeons must be aware of the surgical and patient-specific risk factors that contribute to postoperative hip instability in order to optimize patient safety in both the acute and chronic stages of recovery. Furthermore, the presence of iliopsoas bursitis, degenerative arthritis, and other causes of continued hip pain following closed hip reduction is an important topic that requires further investigation as the incidence of these complications is likely to rise with a growing number of hip arthroscopy procedures. This case emphasizes the need to investigate safer techniques to minimize postoperative complications, avoid dislocation after hip arthroscopy, and successfully manage resulting pathology.
